# Supplementary material for: Enhancing grapevine breeding efficiency through genomic prediction and selection index
Source: G3 (Bethesda). 2024 Feb 24;14(4):jkae038. doi: 10.1093/g3journal/jkae038 (PMC10989862; doi:10.1093/g3journal/jkae038)
Supplement: jkae038_Supplementary_Data [file jkae038_supplementary_data.pdf]

Enhancing grapevine breeding efficiency through genomic prediction and selection index

Supplemental Material

Charlotte Brault[1,2,*], Vincent Segura[1,5], Maryline Roques[1,2], Pauline Lamblin[1,2], Virginie Bouckenooghe[1,2], Nathalie Pouzalgues[3], Constance Cunty[2,3], Matthieu Breil[1,2], Marina Frouin[4], Léa Garcin[2,4], Louise Camps[4], Marie-Agnès Ducasse[2], Charles Romieu[1,5], Gilles Masson[2,3], Sébastien Julliard[4], Timothée Flutre[6], and Loïc Le Cunff[1,2,*]

[1]UMT Geno-Vigne®, IFV, INRAE, Institut Agro Montpellier, Montpellier 34398, France

[2] Institut Français de la vigne et du vin, Pôle National Matériel Végétal, Le Grau du Roi 30240, France

[3]Centre du Rosé, Vidauban 83550, France

[4]Conservatoire du Vignoble Charentais, Institut de Formation de Richemont, Cherves-Richemont 16370, France

[5]AGAP Institut, Univ Montpellier, CIRAD, INRAE, Institut Agro, Montpellier, France

[6]INRAE, CNRS, AgroParisTech, Université Paris-Saclay, GQE—Le Moulon, Gif-sur-Yvette 91190, France

[*]Corresponding authors: charlotte.brault@live.com, loic.lecunff@vignevin.com

List of Figures

Figure S1 Number of offspring for each cross depending on the category 3
Figure S2 Phenotypic structure, results of PCA analysis. 11
Figure S3 Effect of the presence of cross effect in the mixed model on predictive ability 12
Figure S4 Distribution of genotypic values in the validation population 15
Figure S5 Strength and weakness of selected genotypes. 16
Figure S6 Observed and predicted values in across-population prediction 18
Figure S7 Across-population genetic relatedness 19

List of Tables

Table S1 Description of traits, variable name, definition, correspondence with the *Vitis* ontology. 4
Table S2 Summary of variables for raw phenotypic data. 6
Table S3 Summary of fitting information. 8
Table S4 Number of common SNP markers between populations 10
Table S5 Ideotype definition 13
Table S6 Summary of MGIDI output. 14
Table S7 Table of the 15 best individuals for both populations 16

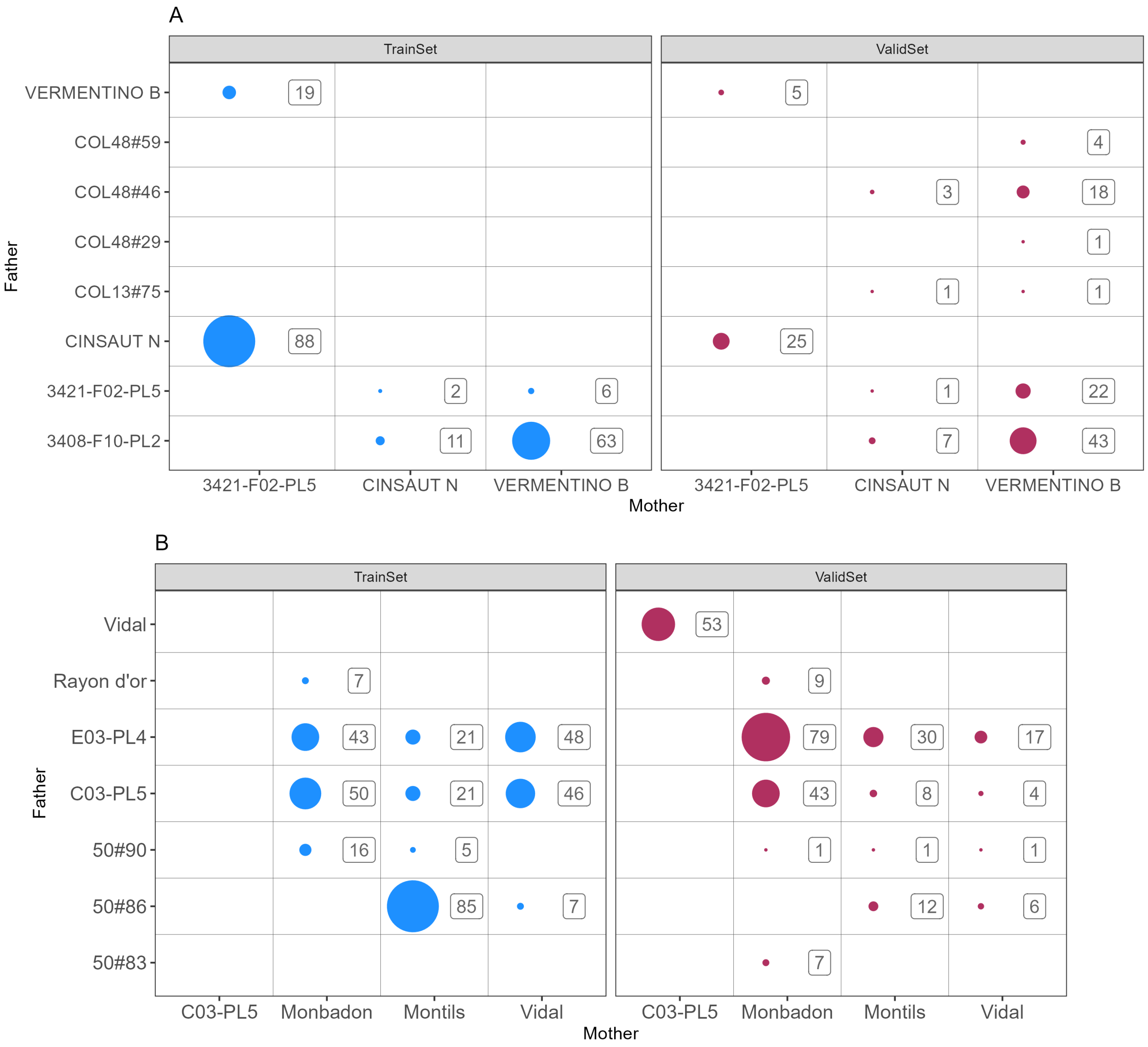

Figure S1: Number of offspring for each cross depending on the category (training set and validation set). A: EDGARR, B: Martell.

Table S1: Description of traits, variable name, definition, correspondence with the *Vitis* ontology.

EDGARR			
Variable	description	VitisOntology_corresp	category
BERRY_ANTHO_meqMV	Berry Anthocyanins content (in meq of malvidin)	NA	Antho
BERRY_pH	Measure of pH on berries	CO_356:1000184	Acid
BER_ALC_C	Potential alcohol content in berries	NA	Sugar
BER_ASCOR_ACID_HPLC_mgl	Concentration of ascorbic acid in berries, measured by HPLC in mg/L	NA	Acid
BER_CAFTA_ACID_HPLC_mg	Concentration of caftaric acid in berries, measured by HPLC in mg/L	NA	Acid
BER_CCOUT_ACID_HPLC_mg	Concentration of cis-coutaric acid in berries, measured by HPLC in mg/L	NA	Acid
BER_COLOR_INT	Color intensitymeasured on berries, determined by the sum of the absorbances at 420, 520 and 620 nm	NA	Color
BER_FRUC_HPLC_g	Fructose concentration in the berries (HPLC, g/l)	NA	Sugar
BER_FRUC_HPLC_mgber	Fructose amount per berry (HPLC, mg/berry)	NA	Sugar
BER_GLUC_HPLC_g	Glucose concentration in the berries (HPLC, g/l)	NA	Sugar
BER_GLUC_HPLC_mgber	Glucose amount per berry (HPLC, mg/berry)	NA	Sugar
BER_GSH_HPLC_mg	Glutathione concentration in the berries (HPLC, mg/l)	NA	Acid
BER_HYDROCIN_mgl	Concentration of hydroxycinnamic acid in berries, measured by the absorbance at 320 nm, in mg/l.	NA	Acid
BER_LIGHT	Perceptual lightness of berries, 0 to 100 for darkness to white (L* parameter).	NA	Color
BER_MAL_HPLC	Malic acid concentration in the berries (HPLC, g/l)	CO_356:1000299	Acid
BER_MAL_HPLC_mgber	Malic acid amount per berry (HPLC, mg/berry)	NA	Acid
BER_PI420	Polymeric pigments measured on berries by the absorbance at 420 nm	NA	Color
BER_PI520	Polymeric pigments measured on berries by the absorbance at 520 nm	NA	Color
BER_RED	Redness measured on berries on a green to red scale (a* parameter).	NA	Color
BER_SHIK_HPLC_g	Shikimic acid concentration in the berries (HPLC, g/l)	NA	Acid
BER_SHIK_HPLC_mgber	Shikimic acid amount per berry (HPLC, mg/berry)	NA	Acid
BER_TART_HPLC_g	Tartaric acid concentration in the berries (HPLC, g/l)	CO_356:1000301	Acid
BER_TART_HPLC_mgber	Tartaric acid amount per berry (HPLC, mg/berry)	NA	Acid
BER_TA_g	Titratable acidity of the berries (g/l H2SO4)	CO_356:1000302	Acid
BER_TCOUT_ACID_mgl	Concentration of trans-coutaric acid on berries, measured by HPLC in mg/L	NA	Acid
BER_TINT	Tint or hue measured on berries, corresponding of the ration between the 420 and 520 nm absorbances.	NA	Color
BER_TOTAL_ANTHO	Total anthocyans measured on berries, by the absorbance at 520 nm	NA	Antho
BER_TPI	Total polyphenol index measured in berries, determined by the absorbance at 280 nm.	NA	Antho
BER_YELLOW	Yellowness measured on berries on a blue to yellow scale (b* parameter).	NA	Color
HARVEST_DOY	Harvest date (Day of year)	CO_356:1000289	Pheno
NB_CLUST_PLANT	Number of clusters per plant	CO_356:1000158	Agro
OD_420	Optical density at 420 nm (yellow color)	NA	Color
OD_520	Optical density at 520 nm (red color)	NA	Color
OD_620	Optical density at 620 nm (blue color)	NA	Color
SBER_W_g	Single berry weight	CO_356:2000144	Agro

Martell			
Variable	Description	VitisOntology_corresp	category
BUD_RELATIVE_CHAS	Bud burst date, relative to the mean bud burst date of Chasselas from plot	NA	Pheno
FERT_GLOB	Number of inflorescences per shoot (all kinds of shoots)	https://cropontology.org/term/CO_356:1000266	Agro
FERT_PRIM	Total number of inflorescences/number of primary shoots	https://cropontology.org/term/CO_356:1000267	Agro
FERT_SPURS	Number of inflorescences per shoot on spurs	https://cropontology.org/term/CO_356:1000265	Agro
FLO_RELATIVE_CHAS	Flowering date, relative to the mean flowering date of Chasselas from plot	NA	Pheno
INT_L	Internode length	https://cropontology.org/term/CO_356:1000093	Vigor
INT_L_45	Internode length of rank 4 and 5 in cm	NA	Vigor
INT_NB	Number of internodes	NA	Vigor
LEAF_ERINOSE_PC	Percentage of erinose symptoms on leaves	NA	Disease
MORPHO_OIV_006	Shoot attitude	https://cropontology.org/term/CO_356:1000198	Techno
MORPHO_OIV_152	Insertion of 1st inflorescence	https://cropontology.org/term/CO_356:1000091	Techno
MORPHO_OIV_204	Bunch density	https://cropontology.org/term/CO_356:1000037	Techno
MORPHO_OIV_240	Berry: ease of detachment from pedice	https://cropontology.org/term/CO_356:1000018	Techno
MUST_ALC_C	Probable alcohol content in must, in %	NA	Vini
MUST_K	Must Potassium concentration	https://cropontology.org/term/CO_356:1000188	Vini
MUST_MAL_gH2SO4l	Concentration of malic acid in must (equivalence g/l of H2SO4)	NA	Vini
MUST_N_AA&NH4	Nitrohen content of the must (sum of N-NH4 and N-amino ac.) in g/l	https://cropontology.org/term/CO_356:1000355	Vini
MUST_TAR_H2SO4	Concentration of tartaric acid in must, expressed in equivalence of H2SO4 in gl	NA	Vini
MUST_TA_H2SO4	Must titratable acidity (equivalence g/l of H2SO4)	https://cropontology.org/term/CO_356:1000293	Vini
MUST_Y_PLANT	Must yield per plant, in mL	NA	Vini
MUST_pH	Must pH	https://cropontology.org/term/CO_356:1000185	Vini
NB_CLUST_PLANT	Number of clusters per plant	https://cropontology.org/term/CO_356:1000158	Agro
SBER_W_g	Single berry weight in gram	https://cropontology.org/term/CO_356:1000215	Agro
SCLUST_W_G	Cluster weight	https://cropontology.org/term/CO_356:1000048	Agro
SHOOT_II_FREQ_0	Frequence of shoots with no internodes	NA	Vigor
SHOOT_L	Primary shoot length	https://cropontology.org/term/CO_356:1000211	Vigor
VER_RELATIVE_CHAS	Véraison date, relative to the mean véraison date of Chasselas from plot	NA	Pheno
VIGOUR_VISUAL	Visual vigour observation in a 1-9 scale (by 2)	NA	Vigor
WOODM_OIV_305	Time of beginning of wood maturity	https://cropontology.org/term/CO_356:1000228	Techno
YIELD_PLANT	Total weight harvest per plant	https://cropontology.org/term/CO_356:1000247	Agro

Table S2: Summary of variables for raw phenotypic data.

EDGARR							
Variable	N	Mean	Std. Dev.	Min	Pctl. 25	Pctl. 75	Max
HARVEST_DOY	403	263.29	24.20	173.00	248.00	280.00	301.00
NB_CLUST_PLANT	343	10.81	7.59	0.00	5.00	15.00	41.00
BER_GSH_HPLC_mg	93	16.26	19.09	0.83	3.22	22.29	95.80
BER_GLUC_HPLC_mgber	179	340.54	79.65	174.90	286.76	376.54	708.15
BER_GLUC_HPLC_g	179	88.47	10.31	45.27	82.16	94.27	129.36
BER_FRUC_HPLC_mgber	179	337.95	78.77	169.33	282.66	372.51	659.49
BER_FRUC_HPLC_g	179	87.83	10.83	43.83	81.44	94.94	126.54
BER_TART_HPLC_mgber	179	30.98	8.39	17.27	24.51	35.90	56.92
BER_TART_HPLC_g	179	8.03	1.62	4.74	6.72	9.24	13.02
BER_MAL_HPLC_mgber	179	51.98	18.59	23.71	38.11	62.73	116.48
BER_MAL_HPLC	179	13.34	3.23	7.08	10.86	15.36	23.30
BER_SHIK_HPLC_mgber	178	0.10	0.10	0.00	0.03	0.13	0.75
BER_SHIK_HPLC_g	178	0.03	0.03	0.00	0.01	0.03	0.17
BER_ASCOR_ACID_HPLC_mgl	101	5.67	2.95	1.11	3.56	7.27	14.80
BER_CAFTA_ACID_HPLC_mg	101	142.56	59.85	38.88	102.19	176.63	312.28
BER_TCOUT_ACID_mgl	101	175.40	99.72	19.05	104.74	225.04	572.03
BER_CCOUT_ACID_HPLC_mg	101	33.94	18.54	7.24	18.93	42.58	100.95
SBER_W_g	267	390.25	125.47	46.54	308.36	467.96	789.90
BER_ALC_C	267	11.79	1.01	7.69	11.34	12.42	14.08
BER_TA_g	265	5.52	1.48	2.90	4.50	6.45	10.40
BERRY_pH	262	3.73	0.23	3.18	3.56	3.90	4.41
BERRY_ANTHO_meqMV	233	70.74	80.00	-8.77	15.34	111.48	503.23
BER_LIGHT	272	63.21	17.61	0.06	50.09	77.08	95.42
BER_RED	272	28.31	18.48	-2.04	13.95	42.52	66.00
BER_YELLOW	272	17.72	9.44	-6.95	10.22	24.43	47.73
OD_420	272	0.80	0.57	0.15	0.48	0.96	7.19
OD_520	272	0.94	0.81	0.07	0.43	1.19	6.85
OD_620	272	0.25	0.27	0.00	0.12	0.31	3.62
BER_COLOR_INT	272	1.99	1.56	0.28	1.04	2.50	17.66
BER_TINT	272	1.11	0.55	0.35	0.68	1.43	3.56
BER_PI420	239	0.54	0.37	0.02	0.32	0.66	3.85
BER_PI520	239	0.28	0.24	0.01	0.15	0.33	2.98
BER_TPI	235	13.11	5.11	4.61	9.64	15.60	43.65
BER_HYDROCIN_mgl	235	9.64	3.60	3.49	6.93	12.10	27.50
BER_TOTAL_ANTHO	235	3.99	4.10	-0.27	1.21	5.89	26.21

Martell							
Variable	N	Mean	Std. Dev.	Min	Pctl. 25	Pctl. 75	Max
BUD_RELATIVE_CHAS	739	9.20	7.11	-2.00	3.00	14.00	50.00
FLO_RELATIVE_CHAS	668	0.97	4.08	-23.00	-1.00	5.00	16.00
VER_RELATIVE_CHAS	661	22.34	10.49	-10.00	11.00	35.00	45.00
WOODM_OIV_305	739	50.10	31.28	0.00	25.00	80.00	100.00
LEAF_ERINOSE_PC	739	0.64	0.92	0.00	0.00	1.00	3.00
MORPHO_OIV_006	738	3.27	1.60	1.00	2.00	5.00	9.00
MORPHO_OIV_152	674	1.92	0.47	1.00	2.00	2.00	3.00
FERT_GLOB	739	5.91	3.48	0.00	3.00	8.00	19.00
FERT_PRIM	739	1.20	0.70	0.00	0.60	1.80	3.80
FERT_SPURS	701	0.57	1.03	0.00	0.00	1.00	6.00
SHOOT_L	739	67.01	24.80	0.00	48.50	82.20	174.20
INT_NB	739	2.47	2.81	0.00	0.00	3.80	48.50
SHOOT_II_FREQ_0	739	0.98	1.38	0.00	0.00	2.00	5.00
INT_L_45	739	5.16	4.77	0.00	2.00	7.18	35.90
INT_L	739	5.26	1.33	0.00	4.40	6.00	11.50
MORPHO_OIV_204	673	2.25	0.75	1.00	2.00	3.00	3.00
YIELD_PLANT	674	1.46	1.18	0.02	0.69	1.92	9.42
NB_CLUST_PLANT	739	7.46	4.59	0.00	4.00	10.00	25.00
SCLUST_W_G	674	175.72	97.45	1.80	110.08	220.07	735.00
MORPHO_OIV_240	672	1.91	0.56	0.50	1.50	2.30	3.50
SBER_W_g	674	3.04	1.23	0.41	2.06	3.87	7.47
MUST_Y_PLANT	674	649.94	636.88	12.00	275.00	860.00	7070.00
MUST_ALC_C	673	11.01	2.36	2.51	9.94	12.55	17.10
MUST_TA_H2SO4	673	4.96	1.91	1.46	3.42	6.20	16.20
MUST_pH	660	3.15	0.19	2.64	3.02	3.26	4.02
MUST_TAR_H2SO4	661	6.19	1.67	0.71	5.10	7.40	11.00
MUST_MAL_gH2SO4l	661	3.04	2.18	0.00	1.46	4.10	16.70
MUST_K	661	1277.36	244.67	788.00	1081.00	1428.00	2156.00
MUST_N_AA&NH4	657	177.48	65.86	27.00	133.00	216.00	557.00
VIGOUR_VISUAL	738	7.42	2.16	1.00	7.00	9.00	9.00

Table S3: Summary of fitting information.

missing.dat: percentage of missing data; ngeno: number of genotypes; fixed.effects: list of fixed effects included; random.effects: list of random effects included; var.geno: genetic variance (genotype + cross effect); var.resid: residual variance, var.tot: total variance, H2: broad-sense heritability; h2: narrow-sense heritability

EDGARR									
trait	missing.dat	ngeno	fixed.effects	random.effects	var.geno	var.resid	var.tot	H2	h2
OD_620	41	169	year	geno, row, col	0.00	0.02	0.03	0.176	0.044
OD_420	41	169	year	geno	0.03	0.12	0.15	0.191	0.059
OD_520	41	169		geno, cross	0.14	0.29	0.55	0.481	0.217
BER_HYDROCIN_mgl	49	152	year	geno, cross	2.95	5.99	12.66	0.527	0.265
BERRY_ANTHO_meqMV	49	150		geno, cross	1,181.89	3,698.13	6,592.34	0.439	0.256
BER_TA_g	42	166	year	geno, row, col	0.61	1.53	2.14	0.284	0.193
BER_FRUC_HPLC_mgber	61	127	year	geno, row, col	0.04	3,692.72	4,362.25	0.000	0.076
BER_GLUC_HPLC_g	61	127	year	geno, row, col	0.21	68.08	111.78	0.003	0.073
BER_GLUC_HPLC_mgber	61	127	year	geno, row, col	190.47	3,836.27	4,557.25	0.047	0.041
BER_LIGHT	41	169	year	geno, cross	58.39	154.87	289.40	0.465	0.271
BER_YELLOW	40	169	year	geno, row, col	5.50	72.92	89.81	0.070	0.239
BER_RED	40	169		geno, cross	61.87	170.49	396.58	0.570	0.314
BER_COLOR_INT	41	169	year	geno, cross	0.32	0.93	1.50	0.377	0.133
BER_TPI	49	152		geno, cross	5.38	14.23	22.78	0.375	0.263
BER_MAL_HPLC	61	127	year	geno	4.18	2.72	6.91	0.606	0.638
BER_MAL_HPLC_mgber	61	127	year	geno	74.12	105.14	179.26	0.414	0.325
BER_TINT	40	169		geno, cross	0.08	0.12	0.31	0.613	0.368
BERRY_pH	43	166	year	geno, row, col	0.01	0.04	0.05	0.249	0.211
BER_PI420	48	151	year	geno, row, col	0.02	0.07	0.09	0.191	0.054
BER_PI520	48	151	year	geno, row, col	0.01	0.02	0.03	0.197	0.091
BER_TOTAL_ANTHO	48	152		geno, cross	3.27	9.81	17.00	0.423	0.260
BER_SHIK_HPLC_g	61	126	year	geno, cross	0.00	0.00	0.00	0.692	0.174
BER_SHIK_HPLC_mgber	61	126	year	geno	0.00	0.00	0.01	0.579	0.574
BER_ALC_C	41	168		geno, row, col	0.13	0.85	1.01	0.136	0.100
BER_TART_HPLC_g	61	127	year	geno, row, col	0.51	1.54	2.64	0.248	0.493
BER_TART_HPLC_mgber	61	127	year	geno, row, col	2.87	42.78	49.21	0.063	0.264
HARVEST_DOY	12	203	year	geno, cross	110.02	400.03	557.32	0.282	0.264
NB_CLUST_PLANT	25	196		geno, row, col	2.78	50.82	58.13	0.052	0.094
SBER_W_g	41	168		geno, cross	3,684.50	11,030.34	16,197.25	0.319	0.185

Martell											
trait	missing.dat	ngeno	semi.quanti	fixed.effects	random.effects	var.geno	var.cross	var.resid	var.tot	H2	h2
DBT_RELATIVE	0	358	FALSE	year	geno	16.76	NA	32.23	49.00	0.342	0.249
ERINO_LEAF_PC	0	358	FALSE	year	geno	0.05	NA	0.49	0.55	0.098	0.141
FERT_COURSON	5	358	FALSE	year	geno	0.20	NA	0.76	0.97	0.211	0.230
FERT_GLOB	0	358	FALSE	year	geno, Cross	1.36	5.29	8.22	12.48	0.341	0.249
FERT_PRIM	0	358	FALSE	year	geno, Cross	0.06	1,925.34	0.32	0.51	0.360	0.263
FLO_RELATIVE	10	349	FALSE	year	geno, col	1.12	NA	7.94	9.47	0.124	0.119
INT_L	0	358	FALSE		geno, row, management	0.20	NA	1.43	1.81	0.122	0.116
MORPHO_OIV_006	0	358	TRUE	year	geno, Cross	0.26	0.03	1.59	1.90	0.164	0.139
MORPHO_OIV_152	9	345	TRUE	year	geno	0.03	NA	0.18	0.21	0.164	0.122
MORPHO_OIV_204	9	348	TRUE		geno, Cross, row, col	0.67	8,304.02	0.02	0.94	0.976	0.986
MORPHO_OIV_240	9	347	FALSE		geno, Cross	0.08	2.90	0.19	0.32	0.406	0.392
MUST_MALIQUE	11	347	FALSE	year	geno, Cross, RPV3	0.93	0.05	1.12	2.84	0.581	0.051
MUST_NASS	11	344	FALSE	year	geno, Cross, row	1,189.98	0.21	2,212.11	4,000.39	0.371	0.417
MUST_pH	11	346	FALSE	year	geno, Cross, col	0.00	5.29	0.01	0.03	0.528	0.363
MUST_PLANT	9	347	FALSE	year	geno	102,540.99	NA	268,445.69	370,986.67	0.276	0.436
MUST_TA	9	347	FALSE	year	geno, Cross, RPV3, row	0.57	0.20	0.76	1.66	0.510	0.936
MUST_TARTRIQUE	11	347	FALSE	year	geno, Cross, row	0.12	1,925.34	1.07	1.55	0.169	0.245
MUST_TAVP	9	347	FALSE		geno	1.40	NA	4.17	5.57	0.252	0.231
NB_CLUST_PLANT	0	358	FALSE	year	geno, Cross	2.78	11.81	14.22	22.28	0.362	0.294
POTASSIUM	11	347	FALSE	year	geno, Cross, row, col	5,927.52	0.16	25,092.09	46,161.80	0.362	0.363
SBER_W_G	9	347	FALSE	year	geno, Cross, RUN_RPV	0.15	0.12	0.47	0.83	0.394	0.397
SCLUST_W_G	9	347	FALSE	year	geno, Cross	2,741.05	0.05	4,912.42	9,578.82	0.487	0.463
SHOOT_II_FREQ_0	0	358	FALSE	year	geno	0.00	NA	0.80	0.80	0.001	0.939
SHOOT_II_L	0	358	FALSE		geno	2.30	NA	20.52	22.81	0.101	0.098
SHOOT_II_NB	0	358	FALSE	year	geno	0.64	NA	3.55	4.19	0.152	0.157
SHOOT_L	0	358	FALSE		geno, Cross, row, management	132.72	0.16	330.83	634.33	0.317	0.354
VER_RELATIVE	11	344	FALSE	year	geno, Cross	25.23	2.90	47.17	84.21	0.440	0.342
VIGOUR_VISUAL	0	358	TRUE	year	geno	0.25	NA	2.73	2.98	0.083	0.071
WOODM_OIV_305	0	358	FALSE	year	geno	27.10	NA	509.85	536.95	0.050	0.061
YIELD_PLANT	9	347	FALSE	year	geno, Cross, col	0.26	114.99	0.85	1.34	0.352	0.371

Table S4: Number of common SNP markers between populations

**Population**	**EDGARR**	**Martell**
**Half-diallel**	3,943 SNPs	2,804 SNPs
**Diversity panel**	6,301 SNPs	4,676 SNPs
**Half-diallel + diversity panel**	3,707 SNPs	2,650 SNPs

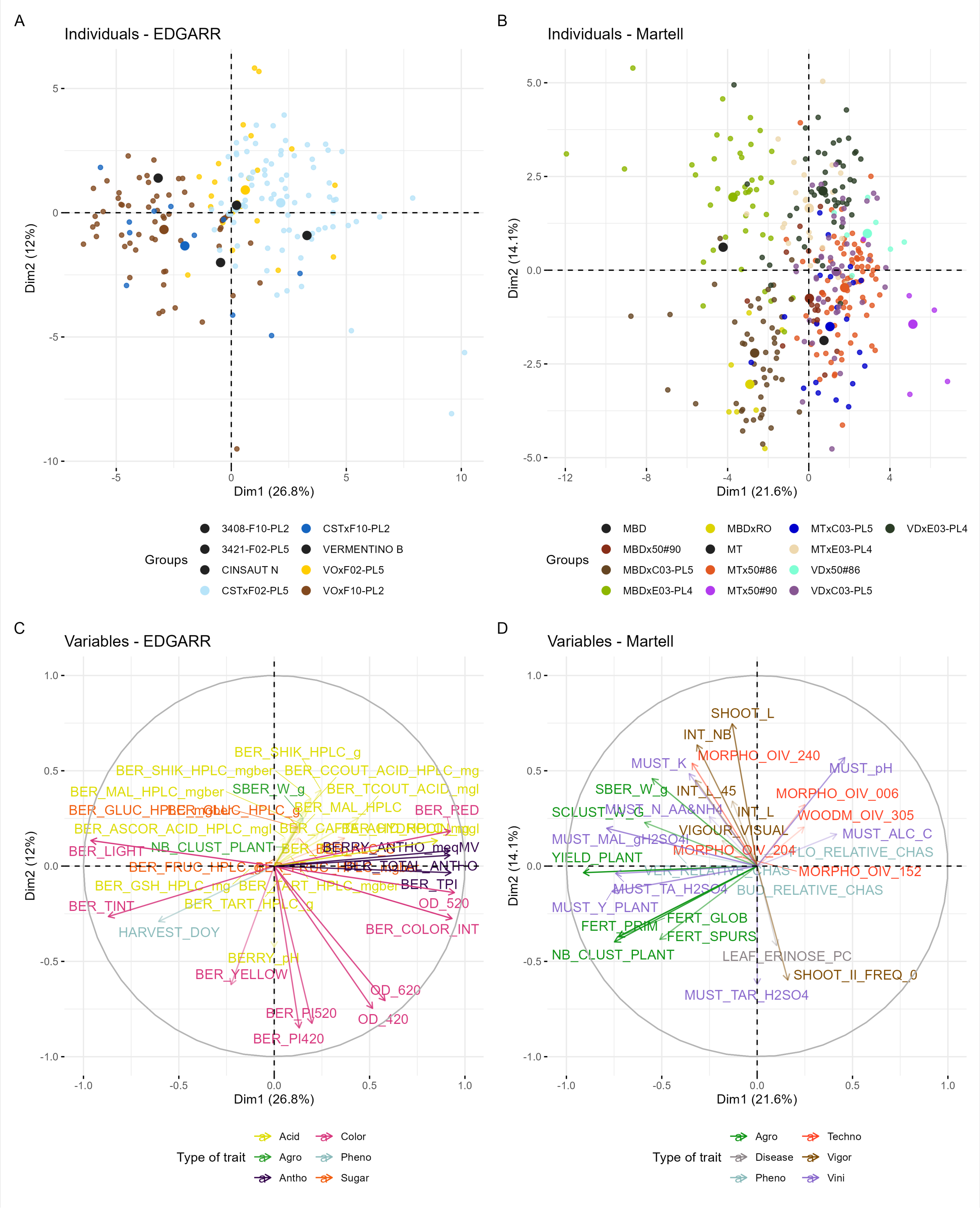

Figure S2: Phenotypic structure, results of PCA analysis. A & B: : projection of individuals for EDGARR and Martell populations, respectively. C & D: plot of variable contributions for the first two axes for EDGARR and Martell populations, respectively

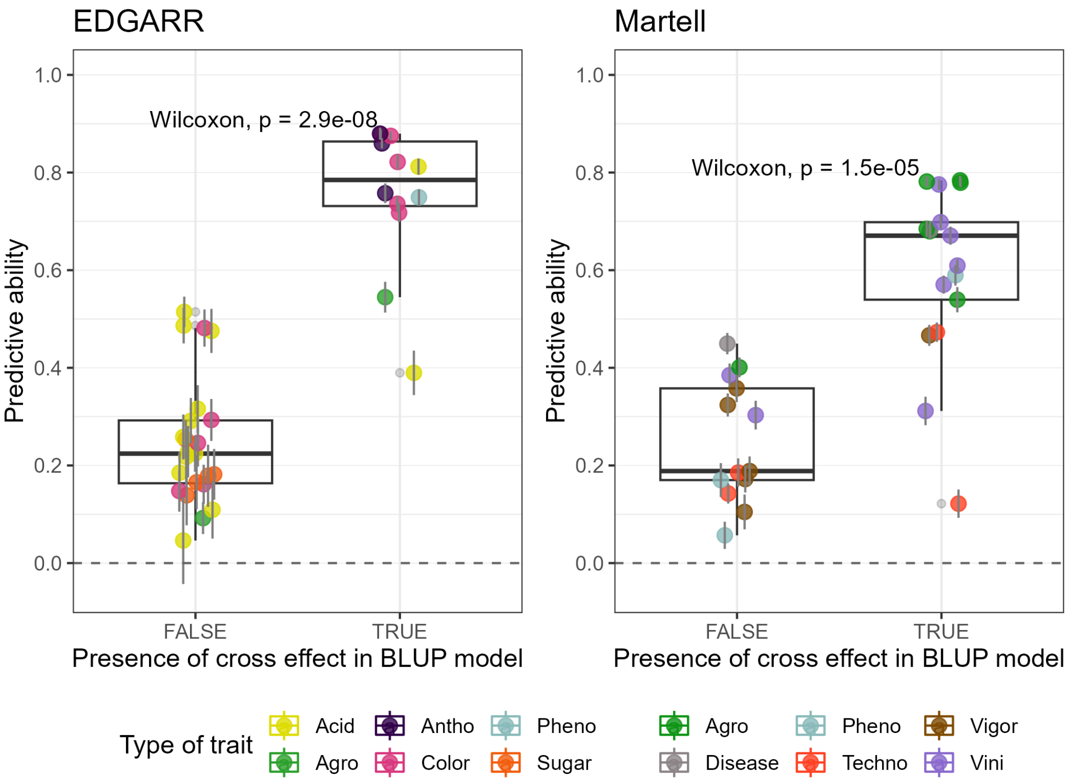

Figure S3: Effect of the presence of cross effect in the mixed model on predictive ability for both populations, for the best method between GBLUP and LASSO. Wilcoxon test was performed between the groups, and a p-value of the test is displayed in the plot.

Table S5: Ideotype definition, the direction column indicates the selection direction, if a numeric value is displayed, an optimum value is sought for this trait.

(a) EDGARR

TRAIT	DIRECTION	weight
**BER_TART_HPLC_g**	9	0.49
**BER_LIGHT**	Max	0.82
**BER_COLOR_INT**	Min	0.72
**HARVEST_DOY**	Max	0.75
**BER_MAL_HPLC**	13	0.32
**BER_TA_g**	7	0.29
**BERRY_pH**	3.5	0.26
**BER_ALC_C**	11	0.25
**BER_TPI**	Min	0.76
**NB_CLUST_PLANT**	Max	0.092
**SBER_W_g**	Max	0.54

(b) Martell

TRAIT	DIRECTION	weight
**MORPHO_OIV_240**	2	0.5
**FERT_GLOB**	Max	0.5
**FERT_PRIM**	Max	0.5
**YIELD_PLANT**	Max	1
**MUST_Y_PLANT**	Max	1
**MUST_MAL_H2SO4**	3	0.5
**MUST_TAR_H2SO4**	Max	1
**MUST_K**	Min	0.5
**SCLUST_W_G**	Max	0.5
**SBER_W_G**	Max	0.5
**MUST_TA_H2SO4**	Max	0.5
**MUST_pH**	Min	0.5

Table S6: Summary of MGIDI output for EDGARR and Martell populations. Factor: closest factor where each trait belongs to, Xo and Xs: mean of the original and selected population, respectively, SD: standard deviation, SDperc: percentage of standard deviation; sense: direction of the trait for the defined ideotype, goal: 0 or 100 if the selection direction has been respected.

EDGARR							
VAR	Factor	Xo	Xs	SD	SDperc	sense	goal
BER_TART_HPLC_g	FA1	1.78	2.08	0.30	16.93	decrease	0
BER_LIGHT	FA1	68.59	70.59	2.00	2.92	increase	100
BER_COLOR_INT	FA1	1.65	1.53	−0.13	−7.69	decrease	100
HARVEST_DOY	FA1	258.46	274.62	16.17	6.26	increase	100
BER_MAL_HPLC	FA2	2.19	3.04	0.85	38.91	decrease	0
BER_TA_g	FA2	1.73	2.57	0.84	48.47	decrease	0
BERRY_pH	FA2	0.17	0.27	0.10	60.32	decrease	0
BER_ALC_C	FA3	0.60	0.46	−0.14	−23.32	decrease	100
BER_TPI	FA3	11.75	8.48	−3.27	−27.82	decrease	100
NB_CLUST_PLANT	FA3	9.86	9.30	−0.56	−5.64	increase	0
SBER_W_g	FA3	368.31	379.24	10.93	2.97	increase	100

Martell							
VAR	Factor	Xo	Xs	SD	SDperc	sense	goal
MUST_TA_H2SO4	FA1	6.92	7.53	0.60	8.73	increase	100
MUST_pH	FA1	3.01	2.94	−0.07	−2.29	decrease	100
YIELD_PLANT	FA1	1.38	1.71	0.34	24.52	increase	100
FERT_PRIM	FA1	1.09	1.55	0.46	41.65	increase	100
FERT_GLOB	FA1	5.37	7.59	2.22	41.44	increase	100
SCLUST_W_G	FA2	168.72	146.22	−22.50	−13.34	increase	0
MUST_TAR_H2SO4	FA2	7.45	8.36	0.91	12.24	increase	100
SBER_W_g	FA2	2.21	2.15	−0.05	−2.40	increase	0
MUST_MAL_gH2SO4l	FA2	2.47	2.50	0.02	0.94	decrease	0
MORPHO_OIV_240	FA3	0.71	0.67	−0.05	−6.37	decrease	100
MUST_K	FA3	1,477.63	1,462.23	−15.40	−1.04	decrease	100

(a) EDGARR

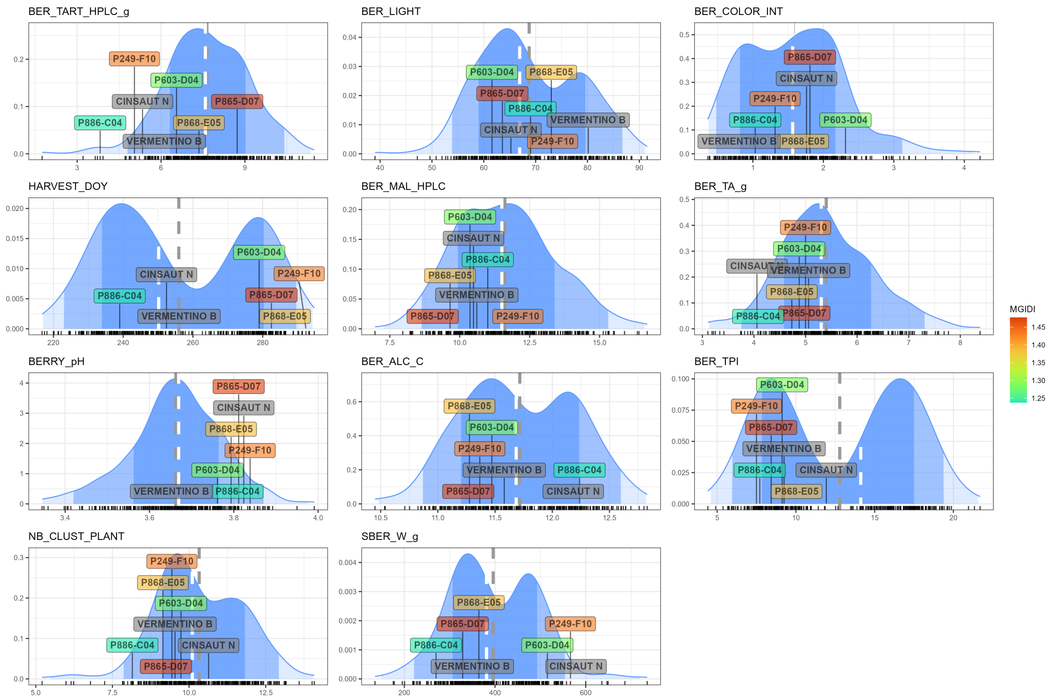

(b) Martell

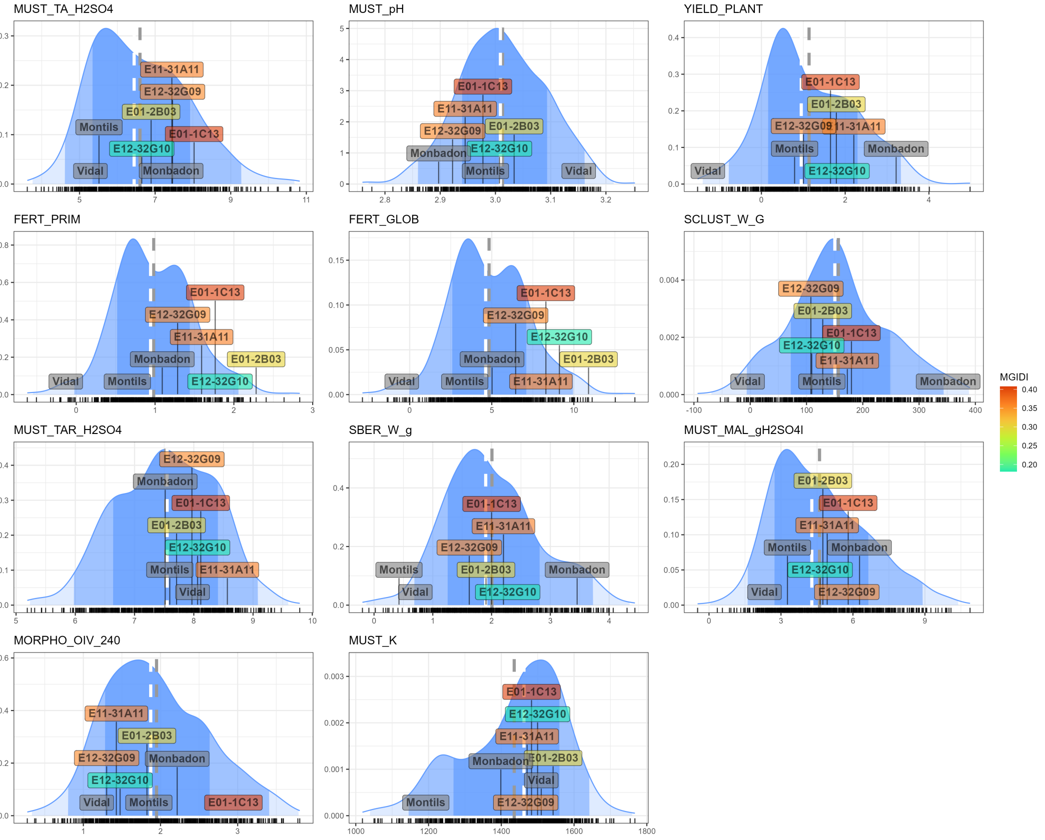

Figure S4: Distribution of genotypic values in the validation population and position of the first five individuals and the emblematic parents. A: EDGARR population, B: Martell population.

Table S7: Table of the 15 best individuals for both populations. MGIDI: multi-trait genotype-ideotype distance index. For the EDGARR population, the LASSO method predicted berry color based on genomic prediction.

EDGARR			
Genotype	Cross	color	MGIDI
P886-C04	Vermentino x COL48-59	white	1.24
P603-D04	Cinsaut x F10-PL2	red	1.29
P868-E05	Vermentino x F10-PL2	red	1.39
P249-F10	Cinsaut x F10-PL2	red	1.43
P865-D07	Vermentino x F10-PL2	red	1.48
P876-D06	Vermentino x COL48-46	white	1.51
P624-A09	Vermentino x F10-PL2	red	1.52
P869-F04	Vermentino x F10-PL2	red	1.59
P886-C07	Vermentino x COL48-59	red	1.65
P596-A09	Cinsaut x F10-PL2	red	1.79
P867-D05	Vermentino x F10-PL2	red	1.79
P862-E05-D	Vermentino x F10-PL2	red	1.87
P869-B07	Vermentino x F10-PL2	white	1.87
P875-D03	Vermentino x COL48-46	red	1.89
P865-B05	Vermentino x F10-PL2	white	1.92

Martell			
Genotype	Cross	MGIDI	recombination
E12-32G10	Monbadon x C03-PL5	0.18	FALSE
E01-2B03	Monbadon x Rayon d'Or	0.30	FALSE
E12-32G09	Monbadon x C03-PL5	0.36	FALSE
E11-31A11	Monbadon x C03-PL5	0.37	TRUE
E01-1C13	Monbadon x Rayon d'Or	0.41	FALSE
E09-26G04	Monbadon x C03-PL5	0.41	FALSE
E59-254B06	C03-PL5 x Vidal	0.49	FALSE
E10-29D10	Monbadon x C03-PL5	0.55	FALSE
E20-60E04	Montils x C03-PL5	0.57	TRUE
E08-25A06	Monbadon x C03-PL5	0.58	FALSE
E11-30C02	Monbadon x C03-PL5	0.58	FALSE
E08-25G04	Monbadon x C03-PL5	0.64	TRUE
E11-32A03	Monbadon x C03-PL5	0.64	FALSE
E11-31A10	Monbadon x C03-PL5	0.67	FALSE
E08-25A03	Monbadon x C03-PL5	0.69	FALSE

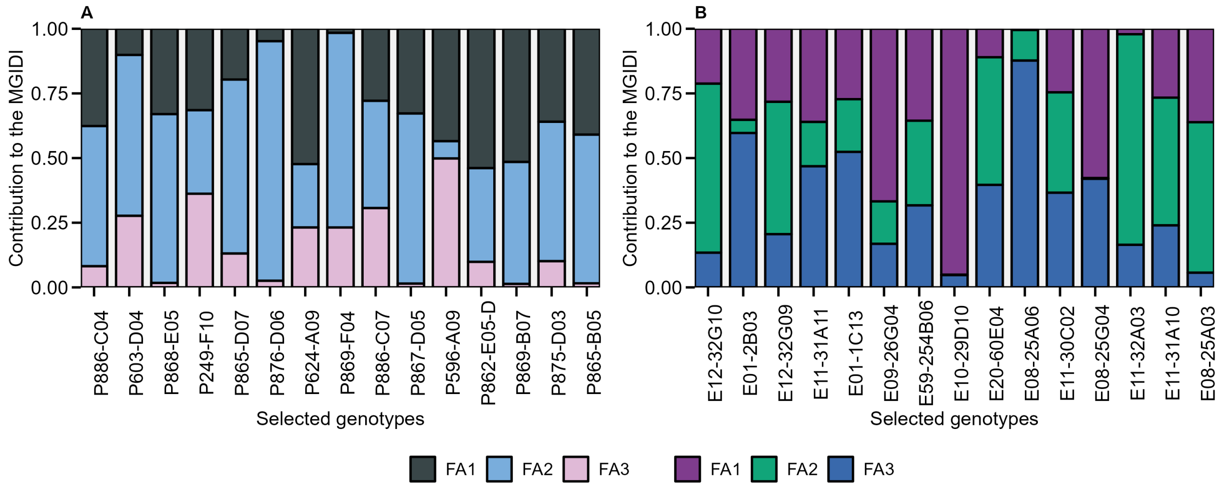

Figure S5: Strength and weakness of selected genotypes. The Y-axis represents the contribution of each factor to the distance to the ideotype. The higher the contribution, the less the genotype is close to the ideotype. Traits contributing to each factor are displayed in Table S6. A: EDGARR population, B: Martell population

(a) EDGARR

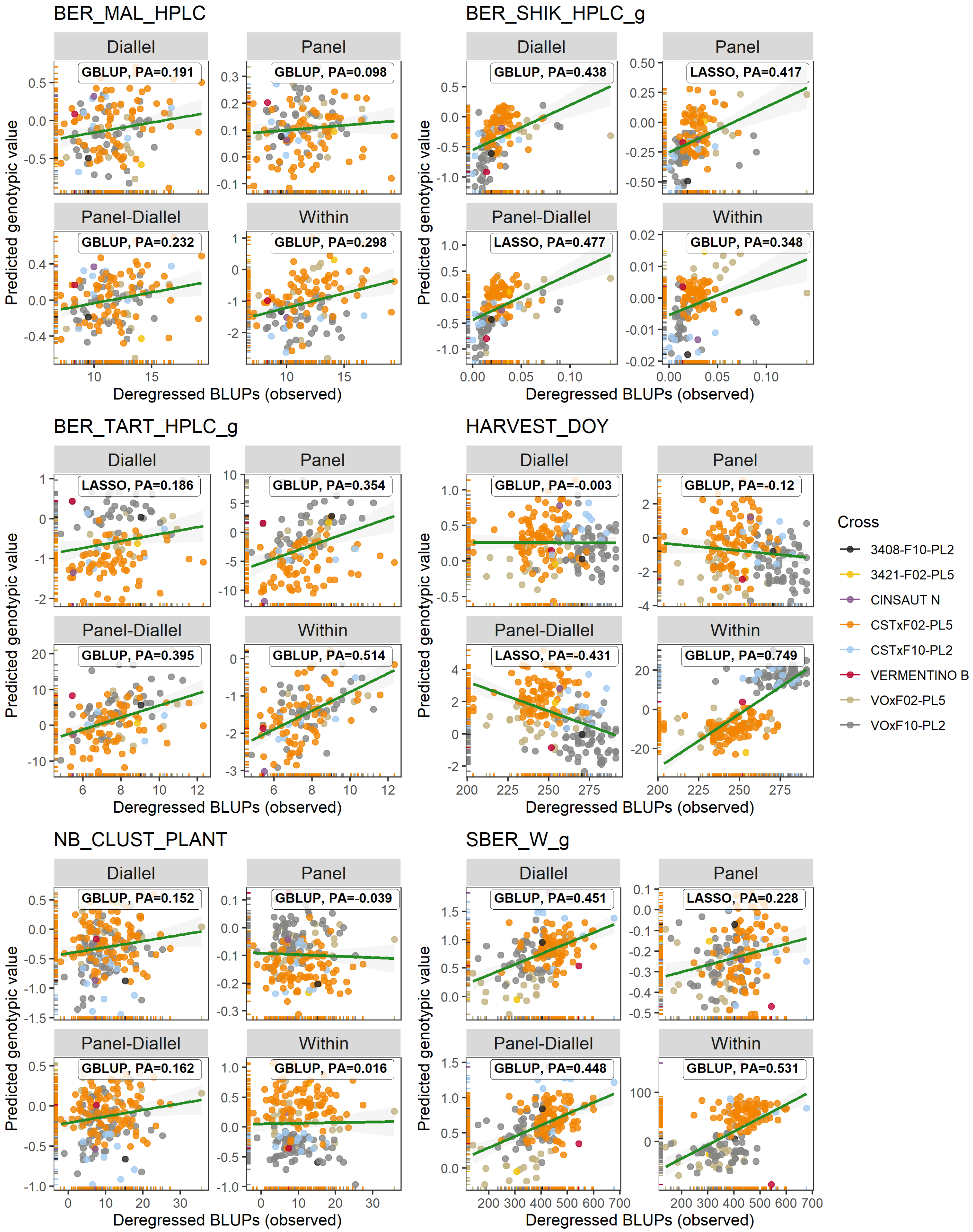
BER_MAL_HPLC
Diallel
GBLUP, PA=0.191
Panel
GBLUP, PA=0.098
Panel-Diallel
GBLUP, PA=0.232
Within
GBLUP, PA=0.298
BER_SHIK_HPLC_g
Diallel
GBLUP, PA=0.438
Panel
LASSO, PA=0.417
Panel-Diallel
LASSO, PA=0.477
Within
GBLUP, PA=0.348
BER_TART_HPLC_g
Diallel
LASSO, PA=0.186
Panel
GBLUP, PA=0.354
Panel-Diallel
GBLUP, PA=0.395
Within
GBLUP, PA=0.514
HARVEST_DOY
Diallel
GBLUP, PA=-0.003
Panel
GBLUP, PA=-0.12
Panel-Diallel
LASSO, PA=-0.431
Within
GBLUP, PA=0.749
NB_CLUST_PLANT
Diallel
GBLUP, PA=0.152
Panel
GBLUP, PA=-0.039
Panel-Diallel
GBLUP, PA=0.162
Within
GBLUP, PA=0.016
SBER_W_g
Diallel
GBLUP, PA=0.451
Panel
LASSO, PA=0.228
Panel-Diallel
GBLUP, PA=0.448
Within
GBLUP, PA=0.531
Predicted genotypic value
Deregressed BLUPs (observed)
Cross
3408-F10-PL2
3421-F02-PL5
CINSAUT N
CSTxF02-PL5
CSTxF10-PL2
VERMENTINO B
VOxF02-PL5
VOxF10-PL2

(b) Martell

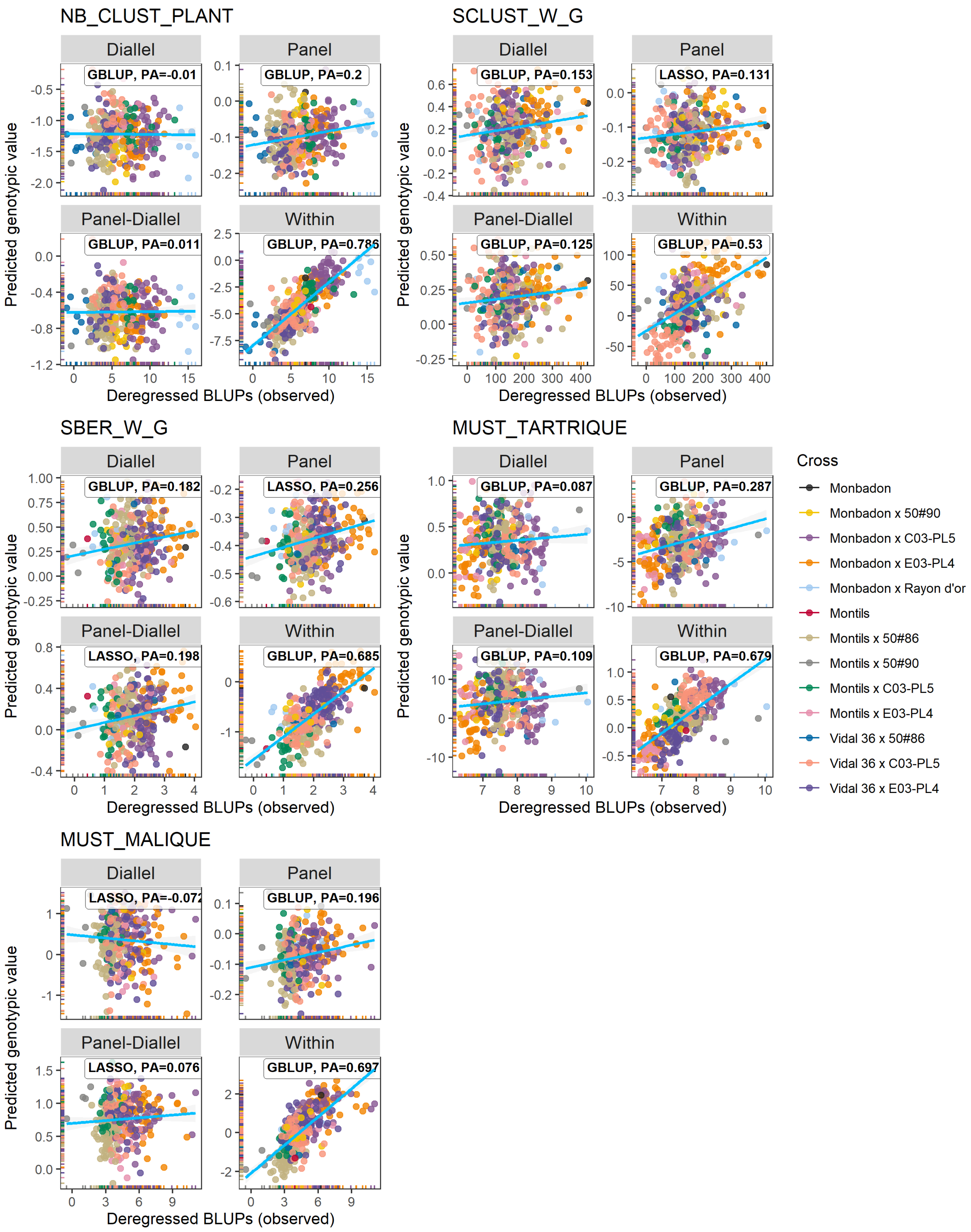

Figure S6: Scatterplot of observed genotypic values vs. predicted value using diverse training population for 6 and 5 traits for EDGARR and Martell population, respectively. The selected best method among LASSO and GBLUP is indicated in each box, as well as predictive ability. For "Within" scenario, a cross-validation within EDGARR or Martell genotypes was applied. A: EDGARR population, B: Martell population.

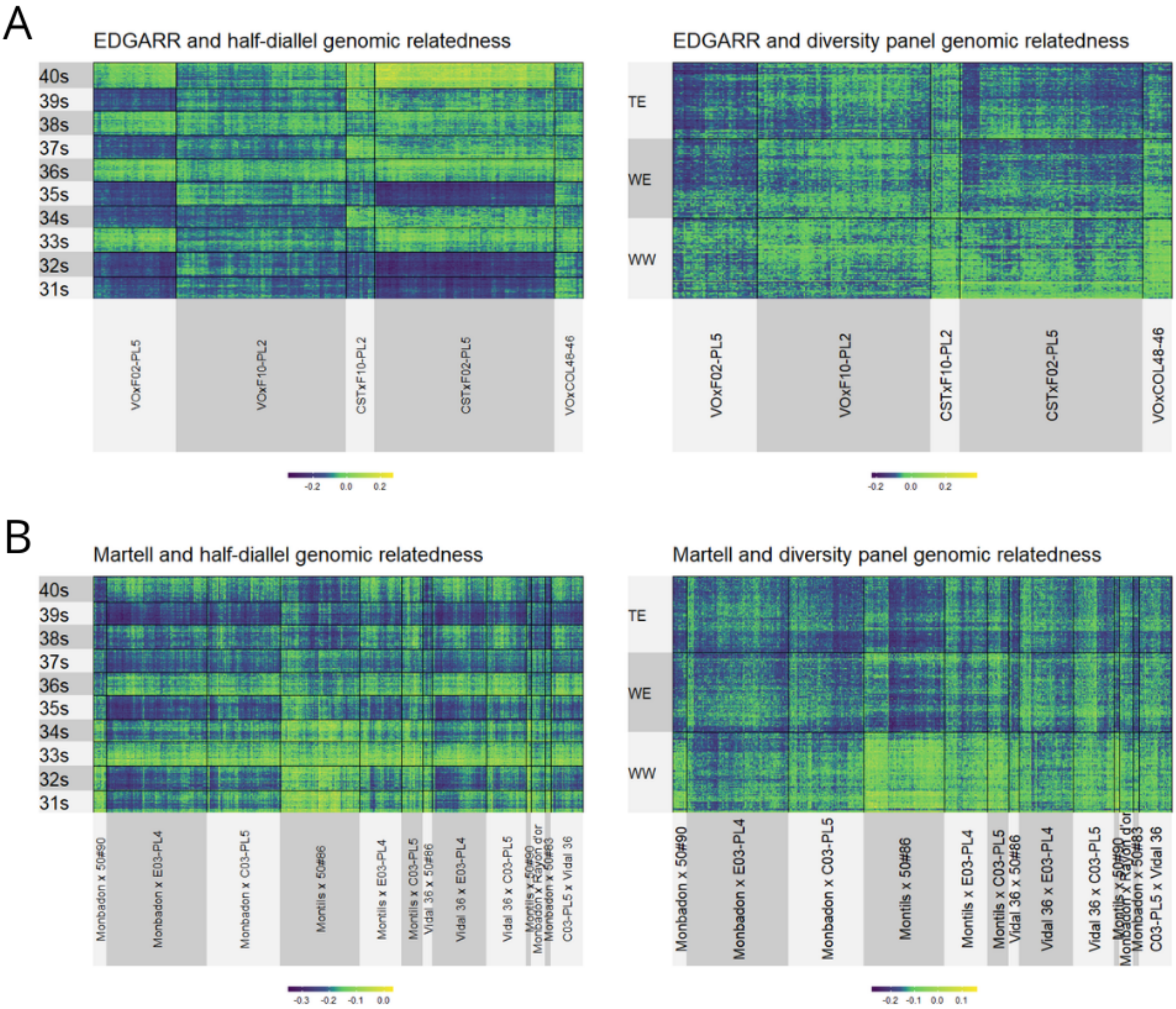

Figure S7: Pairwise additive genetic relatedness between training sets (half-diallel on the left, diversity panel on the right), and validation sets (EDGARR and Martell). Genotypes are structured by half-diallel crosses (31s to 40s), diversity panel subpopulations (WW: Wine West, WE: Wine East, TE: Table East), and EDGARR and Martell crosses. A: EDGARR population, B: Martell population
